# Storage Time and Urine Biomarker Levels in the ASSESS-AKI Study

**DOI:** 10.1371/journal.pone.0164832

**Published:** 2016-10-27

**Authors:** Kathleen D. Liu, Edward D. Siew, W. Brian Reeves, Jonathan Himmelfarb, Alan S. Go, Chi-yuan Hsu, Michael R. Bennett, Prasad Devarajan, T. Alp Ikizler, James S. Kaufman, Paul L. Kimmel, Vernon M. Chinchilli, Chirag R. Parikh, for the ASSESS-AKI Study Investigators

**Affiliations:** 1 Division of Nephrology, Department of Medicine, University of California, San Francisco, San Francisco, CA, United States of America; 2 Department of Medicine, Division of Nephrology and Hypertension, Vanderbilt University Medical Center, Nashville, TN, United States of America; 3 Department of Medicine, Division of Nephrology, Penn State College of Medicine, Hershey, PA, United States of America; 4 Kidney Research Institute, Department of Medicine, University of Washington, Seattle, WA, United States of America; 5 Division of Research, Kaiser Permanente Northern California, Oakland, CA, United States of America; 6 Division of Nephrology, Cincinnati Children’s Hospital Medical Center, Cincinnati, OH, United States of America; 7 Research Service and Renal Section, VA New York Harbor Healthcare System and New York University School of Medicine, New York, NY, United States of America; 8 Division of Kidney, Urologic, and Hematologic Diseases, NIDDK, NIH, Bethesda, MD, United States of America; 9 Department of Public Health Sciences, Penn State College of Medicine, Hershey, PA, United States of America; 10 Section of Nephrology, Department of Medicine, Yale University School of Medicine, New Haven, CT, United States of America; 11 Program of Applied Translational Research, Yale University School of Medicine, New Haven, CT, United States of America; University of Sao Paulo Medical School, BRAZIL

## Abstract

**Background:**

Although stored urine samples are often used in biomarker studies focused on acute and chronic kidney disease, how storage time impacts biomarker levels is not well understood.

**Methods:**

866 subjects enrolled in the NIDDK-sponsored ASsessment, Serial Evaluation, and Subsequent Sequelae in Acute Kidney Injury (ASSESS-AKI) Study were included. Samples were processed under standard conditions and stored at -70°C until analyzed. Kidney injury molecule-1 (KIM-1), neutrophil gelatinase-associated lipocalin (NGAL), interleukin-18 (IL-18), and liver fatty acid binding protein (L-FABP) were measured in urine samples collected during the index hospitalization or an outpatient visit 3 months later. Mixed effects models were used to determine the effect of storage time on biomarker levels and stratified by visit.

**Results:**

Median storage was 17.8 months (25–75% IQR 10.6–23.7) for samples from the index hospitalization and 14.6 months (IQR 7.3–20.4) for outpatient samples. In the mixed effects models, the only significant association between storage time and biomarker concentration was for KIM-1 in outpatient samples, where each month of storage was associated with a 1.7% decrease (95% CI -3% to -0.3%). There was no relationship between storage time and KIM-1 levels in samples from the index hospitalization.

**Conclusion:**

There was no significant impact of storage time over a median of 18 months on urine KIM-1, NGAL, IL-18 or L-FABP in hospitalized samples; a statistically significant effect towards a decrease over time was noted for KIM-1 in outpatient samples. Additional studies are needed to determine whether longer periods of storage at -70°C systematically impact levels of these analytes.

## Introduction

Over the past decade, a major focus of nephrology research has been the identification and measurement of renal tubular injury biomarkers for the diagnosis and risk assessment of acute kidney injury (AKI). These markers include urine kidney injury molecule-1 (KIM-1), neutrophil gelatinase-associated lipocalin (NGAL), interleukin-18 (IL-18), and liver fatty acid binding protein (L-FABP)[1–4]. To date, most studies have used batched enzyme-linked immunoassays (ELISAs) performed on urine samples that had been stored at -70°C for months to years. As these biomarkers have been studied in new contexts where there is heavy reliance on use of samples stored for prolonged periods, including in patients with chronic kidney disease (CKD), the impact of urine processing and duration of storage on biomarker levels has become an area of significant interest. With currently available assays, we and others have demonstrated that the aforementioned four biomarkers appear to be quite robust to a variety of short-term processing conditions [5–7]. For example, compared to immediate storage at -70°, short-term storage at 4°C or room temperature prior to storage at -70°C had minimal impact on biomarker levels aside from IL-18 [5], and lack of centrifugation did not affect biomarker levels.

However, little is known about the impact of duration of storage on biomarker levels, and this is a methodologically challenging problem to study. For example, the impact of duration of storage can be assessed by making repeated measurements on the same sample over an extended time period, but this study design may be affected by assay drift or changes in the assay used over time. Systematic drift in an assay over time may lead to the potentially incorrect conclusion that a biomarker does or does not degrade over time with storage at -70°C. In the case of serum creatinine (sCr), the impact of changes in the assay on the apparent prevalence of CKD has been well described [8, 9]. Another approach that has been taken is to use the intraclass correlation between paired measurements from a single individual to infer differences related to storage time from differences related to biological or assay variability [10].

A novel approach to examine the impact of storage duration on biomarker levels is to perform simultaneous biomarker measurements on samples of varying ages collected rigorously under the same protocol. Subsequently, one can determine whether there is an association of storage time with biomarker levels, after controlling for patient level-factors that might be associated with higher or lower levels of the biomarker. With this approach, assay-level factors (“batch bias”) are well controlled. In the current study, we took this innovative approach to examine the association between storage time and urine biomarkers, using samples from the NIDDK-sponsored ASsessment, Serial Evaluation, and Subsequent Sequelae in Acute Kidney Injury (ASSESS-AKI). ASSESS-AKI is a parallel, matched cohort study of hospitalized patients with and without AKI [11]. The underlying assumption is that, for a well characterized population, the distribution of biomarker values should be similar over variable times of sample storage.

## Materials and Methods

### Participants and Sample Collection

KIM-1, NGAL, IL-18, and L-FABP were measured in urine samples collected from 866 ASSESS-AKI Study participants at the index hospitalization visit and the first outpatient visit approximately 3 months after discharge. To be eligible for the parent ASSESS-AKI study, participants with AKI had to be hospitalized, have blood and urine biospecimens banked within 48 hours of the AKI diagnosis and then return for an outpatient study visit three months after the original index hospitalization. Non-AKI controls had specimens banked during an index hospitalization as well, and were priority matched to cases based on clinical center, age, CKD status, location of AKI episode (intensive care unit versus ward), and pre-existing diabetes and cardiovascular disease). The samples were collected and processed using a standard protocol. Immediately after collection, samples were placed on ice if they were not processed within 30 minutes. The target processing time was within 2.5 hours, but samples could be processed per protocol within 6 hours to make sample collection at home visits feasible. Samples were spun for 10 minutes at 1000g, aliquoted and frozen and shipped to a biorepository for long term storage at –70°C. For each sample, the time that the sample was collected and frozen (the start and end of processing time) were recorded. Institutional Review Boards at all of the relevant institutions approved this work (University of California, San Francisco; Vanderbilt University; Kaiser Division of Research; Yale University; Cincinnati Children's Medical Center; University Of Washington; Pennsylvania State University at Hershey). Written informed consent to participate in this study was obtained from all study subjects.

### Biomarker Analysis

All measurements were made at the ASSESS-AKI core laboratory at Cincinnati Children’s Hospital over approximately 2 weeks using kits from a single lot. All reported coefficients of variation are based on analyses in the performance laboratory. The laboratory and staff measuring the biomarkers were blinded to participant characteristics and storage time.

The urine NGAL ELISA was performed using a commercially available assay (NGAL ELISA Kit 036; Bioporto, Grusbakken, Denmark) that specifically detects human NGAL[12](12)(12). The intra-assay coefficient of variation (CV) was 2.1% and inter-assay CV was 9.1%. The urine KIM-1 ELISA was constructed using commercially available reagents (Duoset DY1750, R & D Systems Inc., Minneapolis, MN) with intra- and inter-assay CV of 2.8 and 7.8%, respectively. Urine IL-18 was measured using a commercially available ELISA kit (Medical & Biological Laboratories Co., Nagoya, Japan) per manufacturer’s instructions (intra- and inter-assay CV = 3.2 and 9.7% respectively). Urine L-FABP was measured using a commercially available ELISA kit (CMIC Co., Tokyo, Japan) per manufacturer’s instructions. Inter- and intra-assay CVs were 3.7% and 10.9%, respectively.

### Analytic Approach

Mixed effects models were used to determine the effect of storage time on log-transformed biomarker levels, controlling for age, gender, diabetes mellitus status, intensive care unit (ICU) stay, clinical center, visit, baseline and peak inpatient serum creatinine levels. These covariates were selected because of their potential influence on biomarker levels and enrollment of individuals with these characteristics might not have been random over time. For example, if the proportion of patients enrolled from study center 1 (with a large critically ill population with more severe AKI) versus study center 2 (with a substantial number of patients with mild AKI) varied over calendar years, biomarker levels might change due to differences in the underlying population (and not due to difference in storage time). Consequently, study center (which would be significantly associated with biomarker levels) was adjusted for in the linear mixed-effects models. A bivariate log-normal model was used to model biomarker levels from both index hospitalization and outpatient time points. Because of the assumption of the bivariate log-normal distribution, this model accounted for measurements that were below the lower limit of detection (e.g. left- censored); we have previously shown that this approach is preferable to data imputation methods to handle data below the lower limit of detection [13]. In a sensitivity analysis to this approach to handling data below the lower limit of detection of the assay, we imputed values below the lower limit of detection for analysis by randomly generating values between zero and the lower limit of detection. All calculations were performed using SAS 9.4 statistical software. A p-value < 0.05 was considered statistically significant.

## Results

Urine biomarker levels (KIM-1, NGAL, IL-18 and L-FABP) were measured from index hospitalization and first outpatient samples from a total of 866 ASSESS-AKI participants. Mean age of participants was 64.0 ± 13.3 years, with the majority being white and male (Table 1). Median storage duration was 17.8 months (25–75% IQR 10.6, 23.6) for in-hospital samples and 14.6 months (IQR 7.3–20.4) for outpatient samples. The proportion of samples with a value below the detection limit of the assay varied by analyte and by visit and was greatest for L-FABP (S1 Table). Analyses were conducted separately for in-hospital versus outpatient biomarker measurements (Table 2) because of challenges in deriving simple confidence intervals which apply to both visit types and the higher median values for samples collected during the index hospitalization for both AKI cases and non-AKI controls (data not shown).

**Table 1:** Baseline Characteristics of ASSESS-AKI Participants (N = 866).

Mean ± SD age	64.0 ± 13.3 years
Women, N(%)	339 (39%)
Race, N (%)	
White/European	730 (84%)
Black/African American	94 (11%)
Other	28 (5%)
Pre-hospitalization CKD, N (%)	296 (34%)
Diabetes mellitus, N (%)	358 (46%)
Hospitalized in ICU, N (%)	384 (44%)
Mean ± SD baseline serum creatinine	1.12 ± 0.47 mg/dL
Mean ± SD peak inpatient serum creatinine	1.60 ± 1.15 mg/dL
Median storage duration (25–75% IQR) of baseline samples	17.8 months (10.6, 23.7)

CKD was defined as an eGFR < 60 mL/min/1.73m^2^, calculated using the baseline serum creatinine and the CKD-EPI equation. No information was systematically available regarding level of proteinuria pre-hospitalization. Baseline serum creatinine was the most recent outpatient serum creatinine available from days 7–365 prior to the index hospitalization.

**Table 2:** Biomarker levels by study visit.

	Index hospitalization Median (25–75% IQR)	3-Month Outpatient Visit Median (25–75% IQR)
KIM-1 (pg/mL)	1384.7 (511.7–3660.8)	714.3 (307.6–1534.6)
NGAL (ng/mL)	36.7 (14.3–93.3)	16.1 (7.5–39.1)
IL-18 (pg/mL)	34.0 (16–73.5)	24.8 (10.8–48.7)
L-FABP (ng/mL)	7.92 (3.5–22.4)	3.04 (2.38–6.71)

In the mixed effects models for outpatient biomarker levels, L-FABP levels were 61% higher in diabetics (95% CI 29%-102%, p < 0.0001), reflecting the established literature suggesting that L-FABP is associated with tubular injury in diabetics in particular [14]. Along the same lines, women had 82% higher levels of NGAL (95% CI 48%-125%, p < 0.0001)[15, 16]. For NGAL, IL-18 and L-FABP, storage time was not significantly associated with biomarker levels in the primary analysis (Table 3), and results were largely consistent for both inpatient and outpatient samples. We did observe that for KIM-1 outpatient samples, each additional month of storage was associated with a 1.7% decrease in levels (95% CI -3% to -0.3%) but this trend was not evident for KIM-1 inpatient samples (95% CI -1.5% to 1.1%).

**Table 3:** Impact of storage time on selected urine biomarker levels.*

	In hospital sample: Storage effect per month (95% CI)	P value	Outpatient sample: Storage effect per month (95% CI)	P value
KIM-1	0.2% (-1.5% to 1.1%)	0.79	-1.7% (-3.0% to– 0.3%)	0.01
NGAL	0.5% (-1.0% to 2.0%)	0.50	-0.4% (-1.8% to 1.1%)	0.64
IL-18	1.1% (-0.1% to 2.4%)	0.07	0.2% (-1.1% to 1.5%)	0.79
L-FABP	0.9% (-0.7% to 2.4%)	0.28	0.7% (-1.0% to 2.4%)	0.41

* Adjusted for age, gender, diabetes status, ICU stay, clinical center, visit, baseline and peak inpatient Cr levels.

Given the large number of samples below the lower limit of detection for some of the analytes, in an alternate analysis we imputed all values below the lower limits of detection. Similar findings were observed (S2 Table). In another sensitivity analysis, we tested the association of tubular injury biomarker levels with albuminuria in the outpatient samples, where albumin and urine creatinine measurements were available. Biomarkers were highly correlated with albumin/Cr ratio (ACR), but including ACR in the model to control for another clinical factor known to associate with biomarker levels did not significantly affect the impact of storage time on biomarker levels (data not shown).

## Discussion

The widespread study of renal tubular injury biomarkers including KIM-1, NGAL, IL-18 and L-FABP as potential biomarkers for acute and chronic kidney disease has resulted in concerns about the impact of sample handling, processing and storage conditions on biomarker levels. For example, it has been established that long-term storage under suboptimal conditions (e.g., storage at -20°C) is associated with degradation of some renal tubular injury biomarkers such as NGAL [17, 18]. However, less is known about the impact of storage time on biomarker levels when samples are stored under more ideal conditions (e.g., at -70°C or lower) for extended periods of time. In the current study, we found a small effect of storage time on levels of urine KIM-1 in samples from an outpatient visit which is stronger than the effect of storage time for other studied biomarkers and for levels of urine KIM-1 in samples from the same participants from an in-hospital visit.

The reasons why this effect would be observed in outpatient, but not in-hospital samples, from the same study subjects are unclear. Like the other three biomarkers, levels of KIM-1 were significantly higher in the in-hospital samples, and it may be that within the ranges of observed values, there was no detectable effect of storage on biomarker levels because levels were higher in the in-hospital samples, and there was more variability between subjects. Our results differ from smaller studies conducted in kidney transplant recipients and healthy volunteers that examined the stability of KIM-1 at 6 and 18 months, respectively [7, 19]. In this regard, our study is different in design and larger than either of these two studies. Furthermore, our storage time analysis extends for up to 36 months. Finally, it is possible that this result is due to type 1 error since multiple comparisons were made as part of the analysis. We chose not to adjust for multiple comparisons since the adjustment can vary widely based on the number of comparisons made. Our results should be interpreted with this in mind, and additional studies are needed to further test the impact of longer-term storage on levels of this analyte.

At present, there is no “gold standard” method to examine the impact of storage time on biomarker levels. A number of studies have taken the approach of re-measuring analytes over time from aliquots of a single sample. However, as a recent study of plasma analytes highlights, when this approach is taken over many years, assay drift over time can have a significant impact on study findings. For example, the population prevalence of CKD varied significantly depending on whether or not a correction factor was applied to sCr [8]. These results have important and direct impact on clinical practice, since inferences about the prevalence of a disease (e.g., CKD) or the clinical utility of a biomarker are often made based on research studies using banked samples. Such banked samples may be evaluated after prolonged periods, and the integrity of such samples, although critical, has typically not been evaluated. In particular, banked biospecimens from established patient cohorts are often used for novel biomarkers in order to develop appropriate cutpoints for use in clinical practice [20, 21]; understanding the stability of the biomarker of interest during long-term storage is critical to determining whether appropriate cutpoints for clinical practice can be validated with banked samples. With regard to urine, the focus in the literature to date has been on measurement of albumin, as well as some other tubular proteins [22–26]. However, a number of these studies stored samples at -20°C, which is now felt to be suboptimal for long term urine storage [27]. For such studies, it is critical to have excellent quality control as well as proficiency samples to ensure that the assays are comparable over time. This is a more important methodologic issue for relatively new biomarker assays such as the ones used in our study, where lot-to-lot differences in assay reagents or calibrators can lead to significant variation in biomarker levels that are unavoidable. While batch-to-batch variation is also more common with these assays than with clinical lab tests, the careful use of control samples can be used to detect this problem. While there are statistical methods that can be applied to make two sets of measurements on the same samples comparable, it is problematic to apply such corrections if analyte stability is uncertain. Analyte stability has also been evaluated by comparing intraclass correlations between samples obtained at different times [10]. However, by nature of the ASSESS-AKI parent study design, the relationship of urine biomarker levels between samples is likely to vary between individuals (e.g., AKI subjects versus non-AKI subjects) and may vary based on the severity of the initial injury (e.g., those who have recovery from AKI versus those who do not).

In the present study, rather than analyzing the same samples longitudinally, we measured biomarker levels in a large, multicenter population and examined the effect of storage time, controlling for differences in the population. Strengths of our analysis include our approach which mitigated an important source of potential bias (i.e., batch bias), the study of multiple biomarkers, as well as our ability to adjust for biological factors known to affect biomarker levels. For example, levels of all four biomarkers were higher at the inpatient hospitalization than the outpatient visit reflecting the effect of AKI. NGAL levels were higher in women, which has previously been shown in other studies [18]. These findings demonstrate the face validity of our analytic approach. Because a greater proportion of values were below the detection limits of the assay during the first outpatient visit (compared to the in-hospital visit), we chose to analyze the impact of storage time for each biomarker by visit type. Since some of the in-hospital samples were non-AKI, non-CKD controls, it is perhaps not surprising that there were values below the lower limit of detection in this analytic sample.

Limitations of our study include the fact that the results are only applicable to the four analytes measured. These biomarkers were prioritized by ASSESS-AKI based on the literature to date in support of their relationship to both AKI and CKD. A second limitation is that our results may not be generalizable to specimens with very prolonged storage times (e.g., decades). Additionally, our samples were stored without protease inhibitors, so we were unable to test the impact of protease inhibitors on the stability of these analytes. However, one can speculate that protease inhibitors should, if anything, improve stability of the analytes measured here. Given the design of the ASSESS-AKI study, a proportion of samples came from non-AKI patients who had normal renal function, and in some cases, a significant proportion of samples had biomarker levels that were below the limit of detection of our assays. However, our results were not different in analyses where those samples with levels below the lower limit of detection were imputed in an alternate strategy which we have shown is less biased than excluding data pairs where one is below the lower limit of detection [13]. Finally, we are unable to exclude lesser degrees of degradation within the bounds of the observed confidence interval.

In summary, among the biomarkers studied (KIM-1, NGAL, IL-18, and L-FABP) and settings examined (hospitalization and ambulatory), we found that only KIM-1 levels from outpatient urine samples were susceptible to change over a period of 36 months at a storage temperature of -70°C. These results highlight the importance of prospective and systematic testing of the impact of storage time on biomarker levels when analyses will be performed on samples collected longitudinally and stored over a period of months to years to ensure greater accuracy of measurement and interpretation.

## Supporting Information

S1 Table Proportion of biomarker measurements below the limit of assay detection. (DOCX)

S2 Table Impact of storage time on selected urine biomarker levels. Levels below the lower limit of detection were imputed.*
(DOCX)
