# Supplementary material for: Storage Time and Urine Biomarker Levels in the ASSESS-AKI Study
Source: PLoS One. 2016 Oct 27;11(10):e0164832. doi: 10.1371/journal.pone.0164832 (PMC5082822; doi:10.1371/journal.pone.0164832)
Supplement: S1 Table — (DOCX) [file pone.0164832.s001.docx]

| Biomarker  (Lower limit of detection) | % Undetectable at Index Hospitalization Visit | % Undetectable at 3-Month Outpatient Visit Follow-Up |
| --- | --- | --- |
|  |  |  |
| KIM-1 (59 pg/mL) | 17.9% | 28.7% |
| NGAL (4.0 ng/mL) | 5.9% | 11.1% |
| IL-18 (12.5 pg/mL) | 17.9% | 28.7% |
| L-FABP (3.0 ng/mL) | 21.5% | 49.8% |
