# Supplementary material for: Storage Time and Urine Biomarker Levels in the ASSESS-AKI Study
Source: PLoS One. 2016 Oct 27;11(10):e0164832. doi: 10.1371/journal.pone.0164832 (PMC5082822; doi:10.1371/journal.pone.0164832)
Supplement: S2 Table — Levels below the lower limit of detection were imputed.* (DOCX) [file pone.0164832.s002.docx]

|  | In hospital sample: Storage effect per month (95% CI) | P value | Outpatient sample: Storage effect per month (95% CI) | P value |
| --- | --- | --- | --- | --- |
|  |  |  |  |  |
| KIM-1 | -0.1% (-1.1% to 1.1%) | 0.82 | -1.7% (-3.0% to -0.3%) | 0.01 |
| NGAL | 0.5% (-1.0% to 2.0%) | 0.50 | 0.4% (-1.9% to 1.1%) | 0.61 |
| IL-18 | 1.2% (-0.1% to 2.6%) | 0.07 | 0% (-1.3% to 1.4%) | 0.94 |
| L-FABP | 0.7% (-0.9% to 2.4%) | 0.40 | 0.6% (1.1% to 2.2%) | 0.51 |

* Adjusted for age, gender, DM status, ICU stay, clinical center, visit, baseline and peak inpatient Cr levels
